# p38 MAPK regulates the Wnt inhibitor Dickkopf-1 in osteotropic prostate cancer cells

**DOI:** 10.1038/cddis.2016.32

**Published:** 2016-02-25

**Authors:** A J Browne, A Göbel, S Thiele, L C Hofbauer, M Rauner, T D Rachner

**Affiliations:** 1 Division of Endocrinology and Metabolic Bone Diseases, Department of Medicine III, Technische Universität Dresden, Dresden, Germany; 2 German Cancer Consortium (DKTK), Partner Site Dresden and German Cancer Research Center (DKFZ), Heidelberg, Germany

## Abstract

The Wnt inhibitor Dickkopf-1 (DKK-1) has been associated with the occurrence of bone metastases in osteotropic prostate cancer by inhibiting osteoblastogenesis. P38 mitogen-activated protein kinase (MAPK) activity is also dysregulated in advanced prostate cancer. However, the impact of p38 MAPK signaling on DKK-1 remains unknown. Inhibition of p38 MAPK signaling in osteolytic PC3 cells by small molecule inhibitors (doramapimod, LY2228820 and SB202190) suppressed DKK-1 expression, whereas activation of p38 MAPK by anisomycin increased DKK-1. Further dissection by targeting individual p38 MAPK isoforms with siRNA revealed a stronger role for MAPK11 than MAPK14 and MAPK12 in the regulation of DKK-1. Moreover, prostate cancer cells with a predominantly osteolytic phenotype produced sufficient amounts of DKK-1 to inhibit Wnt3a-induced osteoblastic differentiation in C2C12 cells. This inhibition was blocked directly by neutralizing DKK-1 using a specific antibody and also indirectly by blocking p38 MAPK. Furthermore, tissue expression in human prostate cancer revealed a correlation between p38 MAPK and DKK-1 expression with higher expression in tumor compared with normal tissues. These results reveal that p38 MAPK regulates DKK-1 in prostate cancer and may present a potential target in osteolytic prostate cancers.

Prostate cancer is the leading cause of cancer-related death in men, second only to lung cancer.^1^ The survival rate for local and regional stages at diagnosis is close to 100% after 5 years; however, this drops to <30% in the case of advanced disease at diagnosis where the cancer has spread to distal lymph nodes, the bones or other organs.^2^ Bone metastases, in particular, exhibit in an increased state of morbidity characterized by skeletal-related events, including pathological fractures and spinal cord compression, which considerably reduce a patient's quality of life.^3, 4^ Bone metastases can generate two types of characteristic lesions; osteoblastic (osteosclerotic), where bone formation is increased (albeit of low quality bone) and osteolytic, where bone loss and destruction are increased. In the clinical setting, histological examinations often show that metastatic lesions arising from solid tumors are heterogeneous.^5^ Although maintaining a degree of heterogeneity, prostate cancer metastases have traditionally been observed to form predominantly osteoblastic lesions.^6^ Despite this, evidence suggests that osteolytic activity is required to precondition bone tissue during the development of prostate cancer bone metastasis.^7, 8^

One key feature of osteolytic activity in bone metastases is an impaired function of the osteoblasts, caused by tumor-derived factors. Among them, the Wnt signaling inhibitor Dickkopf-1 (DKK-1) is considered to have a major role. Wnt signaling regulates osteoblast differentiation and function and is therefore important for bone homeostasis.^9^ Therefore, DKK-1 as a Wnt inhibitor negatively regulates osteoblast differentiation.^10^ Although the role of DKK-1 in cancer remains controversial with claims of both tumor-suppressor and promotor roles depending on the cancer type,^11, 12, 13, 14, 15^ it has been convincingly demonstrated that elevated levels are responsible for the induction of osteolytic lesions in bone-seeking cancers such as multiple myeloma and breast cancer.^16, 17, 18, 19^ Furthermore, we have previously shown that DKK-1 is elevated in the serum of prostate cancer patients and high levels of serum DKK-1 were associated with a poorer prognosis.^20^ In addition, elevated levels of DKK-1 in prostate bone metastases have also been associated with a poorer survival.^21^

P38 mitogen-activated protein kinases (MAPKs) are activated by a variety of environmental insults and inflammatory cytokines, controlling numerous cell functions, including cell cycle, apoptosis and proliferation. p38 MAPK comprises four unique isoforms (p38*β*/MAPK11, p38γ/MAPK12, p38δ/MAPK13 and p38α/MAPK14) sharing varied degrees of sequence homology and activity. MAPK14 and MAPK11 are the two most discussed in cancer research to date, being the closest related in structure, activity and sensitivity to inhibitors.^22^

It has been shown that activation of p38 MAPK in multiple myeloma, promotes the destructive, osteolytic phenotype of bone metastases by interfering with the coupling of osteoblast and osteoclast differentiation and recruitment. Furthermore, p38 MAPK was identified as a regulator of DKK-1 expression.^23^ In prostate cancer, p38 MAPK activity is also dysregulated and reports present both tumorigenic and tumor-suppressor roles.^24, 25, 26, 27^ In the context of malignant bone disease, MAPK11 in particular has been shown to augment osteoclastogenesis in breast cancer-induced bone resorption.^28^

To date, the regulatory role of DKK-1 by p38 MAPK in prostate cancer remains unknown. Here, we hypothesize that signaling of p38 MAPK regulates DKK-1 expression in prostate cancer, supporting the osteolytic phenotype by impairing osteoblastogenesis. The specific aims of this study were to determine if p38 regulates DKK-1 in prostate cancer and to assess if targeting p38 MAPK may have positive effects against DKK-1-inhibited osteoblastogenesis.

## Results

### DKK-1 is highly expressed in osteolytic PC3 cells and inhibits osteoblastic differentiation

**Figure 1:**
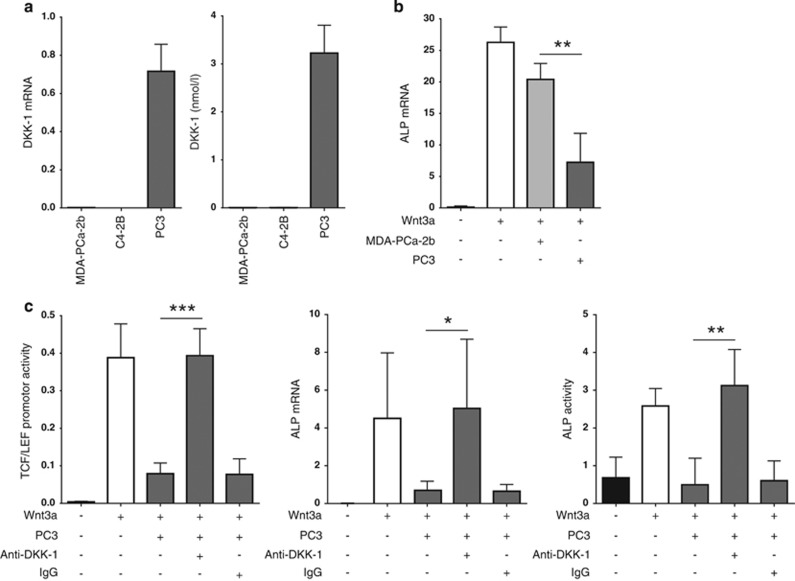
DKK-1 is highly expressed in osteolytic prostate cancer cells and inhibits Wnt3a-induced osteoblastogenesis in C2C12 cells. (**a**) Total mRNA and secreted protein levels of DKK-1 were measured by qRT-PCR analysis and ELISA respectively in prostate cancer cell lines. (**b**) Supernatants of prostate cancer cell lines MDA-PCa-2b and PC3 where harvested after 48 h. C2C12 cells underwent differentiation in the presence of Wnt3a media (10%), 5% FCS DMEM/F-12 (75%) and prostate cancer supernatant (15%) for 72 h. Ten percent L-cell media were used in the control conditions. The mRNA levels of the osteoblastic marker ALP were assessed by qRT-PCR. (**c**) C2C12 cells were transfected with the TCF/LEF Wnt promoter and treated in the presence of Wnt3a medium with PC3 supernatant and 1 *μ*g/ml anti-DKK-1 or 1 *μ*g/ml IgG goat for 24 h before lysis and assay. Activation of Wnt signaling was detected by measuring luciferase activity. ALP mRNA expression levels by qRT-PCR and ALP activity (arbitrary units) by enzymatic assay were assessed following the same experimental conditions as listed in (**b**). For prostate cancer cell lines and C2C12 experiments, mRNA expression data shown are normalized to beta-actin and murine beta-actin, respectively. Results are shown as the mean±S.D. (*P<0.05; **P<0.01, ***P<0.001) and *N*=3

DKK-1 mRNA expression and protein secretion were assessed in PC3, MDA-PCa-2b and C4-2B prostate cancer cells lines. DKK-1 was barely detectable in MDA-PCa-2b and C4-2B cell lines, which are known to induce osteoblastic and mixed lesions *in viv*o. In contrast, the osteolytic PC3 cells displayed a strong baseline expression of DKK-1 levels, measured by RNA expression and protein levels in cell supernatants (Figure 1a). To verify the suppressive effect of DKK-1 on osteoblastogenesis,^29, 30, 31^ we chose the C2C12 cell line, which can be induced along the osteoblastic lineage in the presence of Wnt3a. Prostate cancer supernatants from the osteolytic PC3 cells potently suppressed the Wnt3a-mediated induction of osteoblastogenesis as seen by decreased levels of alkaline phosphatase (ALP) expression. Supernatants collected from the MDA-PCa-2b had little to no suppressive effect (Figure 1b). Following Wnt3a exposure, Wnt activity in C2C12 cells was increased >100-fold with respect to the L-cell control, as seen by TCF-LEF reporter assay analysis. A strong antagonism of Wnt signaling was then apparent in the presence of PC3 supernatant, which was also reflective in the expression and activity of the osteoblastic marker ALP. To prove that these effects were mediated by PC3-derived DKK-1, a monoclonal antibody against DKK-1 was introduced to the culture conditions. This resulted in a complete reversal of the observed suppressive effect of DKK-1 on Wnt3a-induced osteoblastogenesis in C2C12 cells (*P*<0.05; Figure 1c). The same trends of Wnt3a induction and DKK-1 suppression were also valid for the Wnt target gene osteoprotegerin (OPG) (Supplementary Figure S1).

### Inhibition and activation of p38 MAPK signaling regulates DKK-1

**Figure 2:**
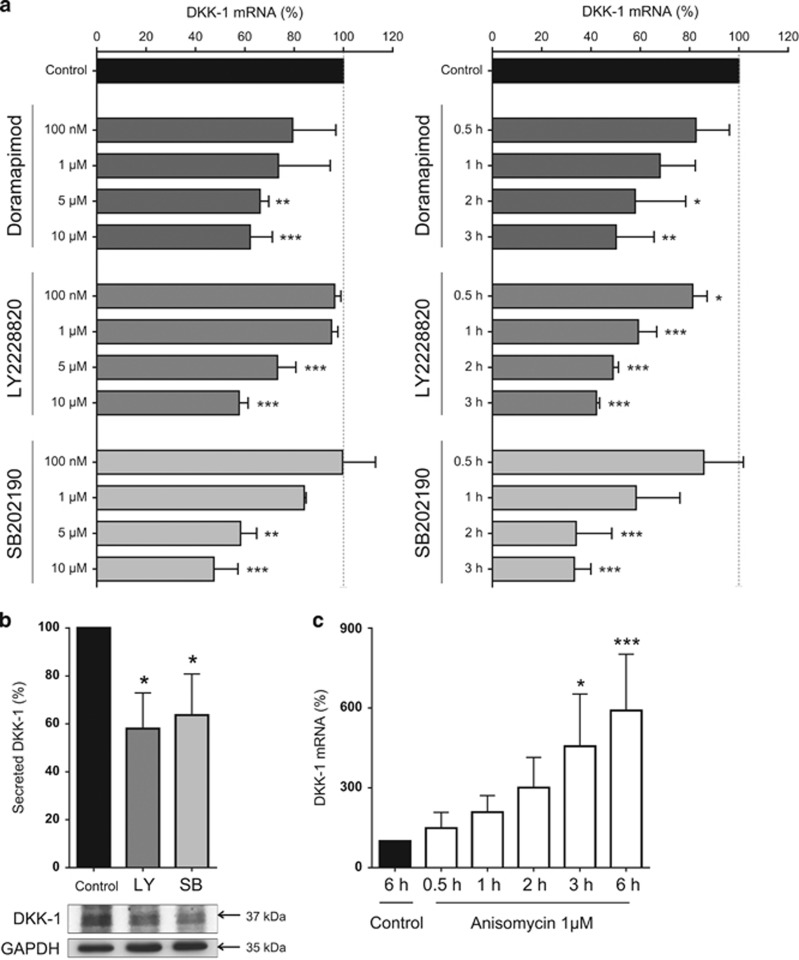
Inhibition and activation of p38 MAPK signaling regulates DKK-1. (**a**) PC3 cells were treated for up to 3 h with small molecule inhibitors of p38 MAPK signaling; doramapimod, LY2228820 and SB202190. The most effective concentration in suppressing DKK-1 expression (10 *μ*M) was used to assess the expression of DKK-1 mRNA in a time-dependent manner. Time points shown are in hours. (**b**) In PC3 cells, total DKK-1 protein and secreted protein levels were assessed for LY2228820 (LY) and SB202190 (SB) after 6 h. (**c**) PC3 cells were treated with the p38 MAPK signaling activator anisomycin for increasing time points from 30 min to 6 h and DKK-1 mRNA expression was assessed. All mRNA expression data of *N*=3 are shown as a percentage of the control untreated group and results are shown as the mean±S.D. (*P<0.05; **P<0.01, ***P<0.001)

**Figure 3:**
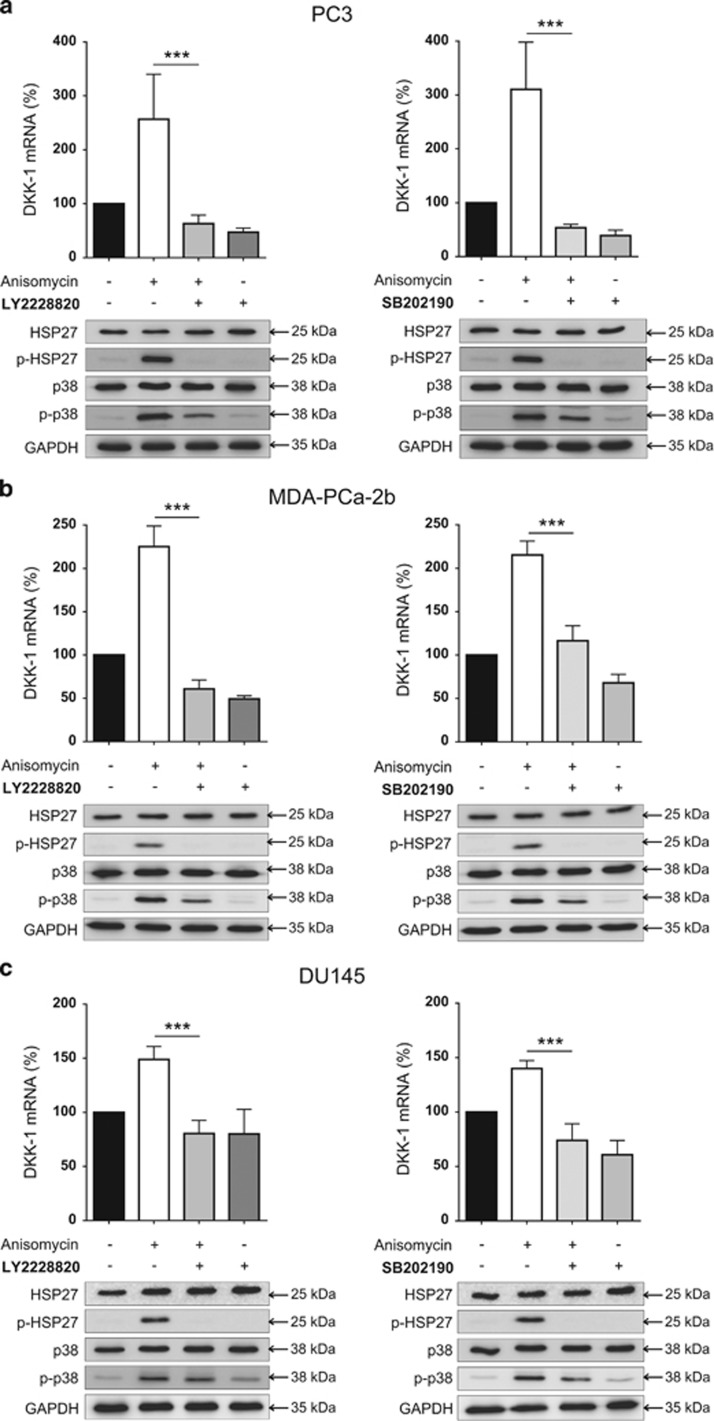
p38 MAPK signaling regulates DKK-1 in osteolytic and osteoblastic prostate cancer cells. PC3 (**a**), MDA-PCa-2b (**b**) and DU 145 cells (**c**) were treated with anisomycin (2 h post culture medium change for 1 h), anisomycin in combination with either LY2228820 or SB202190 (anisomycin after 2 h post culture medium change for 1 h and LY and SB for 3 h immediately post culture medium change) and each selected inhibitor individually (for 3 h). DKK-1 mRNA expression and both total and phosphorylated protein levels of p38 MAPK and HSP27 were assessed after 3 h by western blot. All mRNA expression data of *N*=3 are shown as a percentage of the control untreated group and results are shown as the mean±S.D. (***P<0.001)

To determine whether or not DKK-1 is regulated by p38 MAPK in prostate cancer cells, PC3 cells were treated with the p38 inhibitors doramapimod, LY2228820 and SB202190. All inhibitors induced a significant suppression of DKK-1 mRNA expression in a time- and dose-dependent manner, with the strongest suppression of 50% or more achieved by all inhibitors at a dose of 10 *μ*M and after 3 h of inhibitor treatment (Figure 2a). This suppression of DKK-1 by p38 MAPK inhibitors was also apparent in another prostate cancer cell line, DU145 (Supplementary Figure S2). When analyzing the two most potent inhibitors (LY2228820 and SB202190), decreased mRNA expression of DKK-1 also translated to reduced DKK-1 protein and secreted protein levels as detected by western blot and enzyme-linked immunosorbent assay (ELISA; Figure 2b). In line with these findings, anisomycin, which is known to activate p38 MAPK, resulted in a rapid and potent threefold increase in DKK-1 expression at a dose of 1 *μ*M after 2 h (Figure 2c). At the protein level, western blot analysis verified the activation of p38 MAPK signaling by showing an increased phosphorylation of p38 MAPK and the downstream target heat shock protein 27 (HSP27). Of note, the increase in DKK-1 expression by anisomycin was prevented by LY228820 and SB202190, and the phosphorylation of p38 MAPK and HSP27 was visibly reduced. This finding further indicates that the effect of anisomycin on DKK-1 is directly mediated by p38 MAPK (Figure 3a). This experimental approach was also repeated for the osteoblastic MDA-PCa-2b (Figure 3b) and mixed osteoblastic/osteolytic DU145 (Figure 3c) cell lines. In both cell lines, an increased DKK-1 mRNA expression was apparent upon p38 activation using anisomycin, which could be suppressed by both p38 MAPK inhibitors. The assessment of secreted DKK-1 protein following anisomycin treatment was not valid because of impaired cell vitality in all cell lines and the general inhibition of protein synthesis provoked by anisomycin.

### MAPK11 is the most significant regulator of DKK-1 mRNA expression in the p38 MAPK family

**Figure 4:**
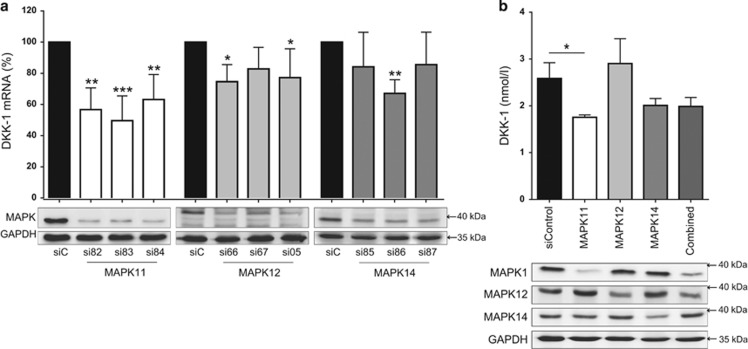
MAPK11 is the most significant regulator of DKK-1 mRNA expression in the p38 MAPK family. (**a**) PC3 cells underwent siRNA transfection targeting p38 MAPK family members, MAPK11, MAPK12 and MAPK14. For each MAPK member, three individual siRNAs were assessed. Twenty-four hours post transfection, DKK-1 mRNA expression was analyzed by qRT-PCR. Confirmation of MAPK knockdown is shown at the protein expression level as assessed by western blot. The three p38 MAPK blots (MAPK) are arranged linearly and correlate to each specific MAPK isoform antibody, the name of which appears below each respective blot. (**b**) The siRNA most effective at suppressing DKK-1 for each MAPK family member (MAPK11; si83, MAPK12; si66 and MAPK14; si86) was selected to assess for any further suppression when transfected in combination. Secreted DKK-1 protein levels in PC3 cell supernatant were assessed 48 h post transfection by ELISA. Western blots of MAPK family members were also performed to confirm knockdown at the level of protein expression. mRNA expression data of *N*=4 are shown as a percentage of the control, scrambled siRNA-transfected group and results are shown as the mean±S.D. (*P<0.05; **P<0.01, ***P<0.001)

To define the individual contribution of the p38 MAPK isoforms to the observed findings, we assessed the roles of MAPK11, MAPK12 and MAPK14 using siRNA transfection in PC3 cells. The efficacy and the specificity of the knockdown were evaluated at mRNA and protein level. Three siRNA sequences were used per p38 MAPK isoform and a sufficient knockdown was achieved for all siRNAs (Supplementary Figure S3). These knockdowns resulted in a suppression of DKK-1 in all three sequences for MAPK11, two sequences for MAPK12 and one sequence for MAPK14 (Figure 4a). It must be noted here that MAPK11 achieved the strongest knockdown at the protein level and this may impact the magnitude of effect on DKK-1 expression compared with the other MAPK isoforms. For each p38 MAPK isoform, the siRNA sequence with the greatest suppression of DKK-1 mRNA was selected and transfected in combination. Combination knockdown did not result in enhanced DKK-1 suppression and the individual knockdown of MAPK11 maintained the strongest correlation with DKK-1 suppression at mRNA level (Supplementary Figure S4). Secreted DKK-1 protein in PC3 supernatant was measured 48 h post transfection by ELISA. Here, DKK-1 protein levels were reduced by 33% for MAPK11 and by 27% for MAPK14. No reduction was seen for MAPK12 (+ 6%) and there was no amplified suppression in the combined knockdown (Figure 4b).

### Suppression of PC3-derived DKK-1 by targeting p38 rescues osteoblastogenesis in C2C12 cells

**Figure 5:**
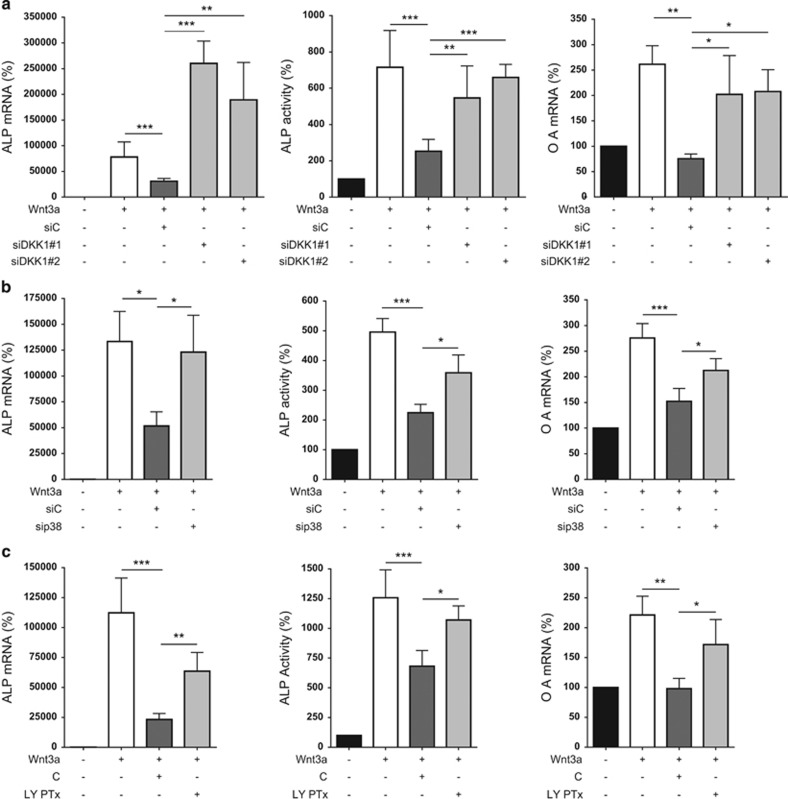
Regulating PC3-derived DKK-1 has reversal effects on suppressed osteoblastogenic differentiation of C2C12 cells. (**a**) Transient knockdown of DKK-1 in PC3 cells was achieved using two different siRNAs. The supernatant of transfected cells was removed and supplemented with fresh medium 24 h post transfection. Supernatants used in experiments were then collected 48 h later. Control siRNA (siC) and two DKK-1 siRNA PC3 supernatant (siDKK-1#1 and #2) (15%) were used to treat C2C12 cells in combination with Wnt3a-containing L-cell media (10%) and 5% FCS DMEM/F-12 (75%) for 72 h. Ten percent L-cell was used in the control conditions and 200 ng/ml BMP-2 was supplemented to all conditions. ALP and osteoactivin (denoted OA) mRNA expression levels were then assessed by qRT-PCR and ALP activity by enzymatic assay. (**b**) DKK-1 expression was suppressed indirectly by combination knockdown of p38 MAPKs in PC3 using siRNAs directed against MAPK11, MAPK12 and MAPK14. PC3 supernatant was harvested and used to treat C2C12 cells as previously detailed (siC=si control RNA and sip38=siRNA combination of the three p38 MAPK isoforms). Assessment of ALP mRNA expression, ALP activity and osteoactivin mRNA expression was then performed. (**c**) DKK-1 expression was suppressed using the p38 MAPK inhibitor LY2228820. PC3 cells were pre-treated with the inhibitor (10 *μ*M) for 6 h before performing a fresh medium change and collecting supernatant 18 h later (LY PTx). These supernatants were then used to treat C2C12 cells as detailed previously (C=control PC3 supernatant). ALP mRNA expression, ALP activity and osteoactivin mRNA expression levels were then analyzed. mRNA expression data of *N*≥3 are shown as a percentage of the control L-cell treatment and results are shown as the mean±S.D. (*P<0.05; **P<0.01, ***P<0.001)

C2C12 cells were treated with conditioned PC3 supernatant where DKK-1 expression had been knocked down by siRNA transfection. ALP mRNA expression, ALP activity and osteoactivin expression levels were all suppressed in the presence of control siRNA-transfected PC3 supernatant and rescued with siDKK-1-transfected PC3 supernatant (Figure 5a). Supernatants of PC3 cells, where p38 MAPK was knocked down, resulted in a rescue effect on the osteoblast markers when compared with control siRNA-transfected PC3 supernatant (Figure 5b). Finally, PC3 cells were pre-conditioned with the p38 inhibitor LY2228820. Here, applying control PC3 supernatant significantly suppressed expression and activity of the osteoblast markers, which were partially rescued when replaced with inhibitor-treated PC3 supernatant (Figure 5c).

### p38 MAPKs and DKK-1 are correlated in human prostate cancer

**Figure 6:**
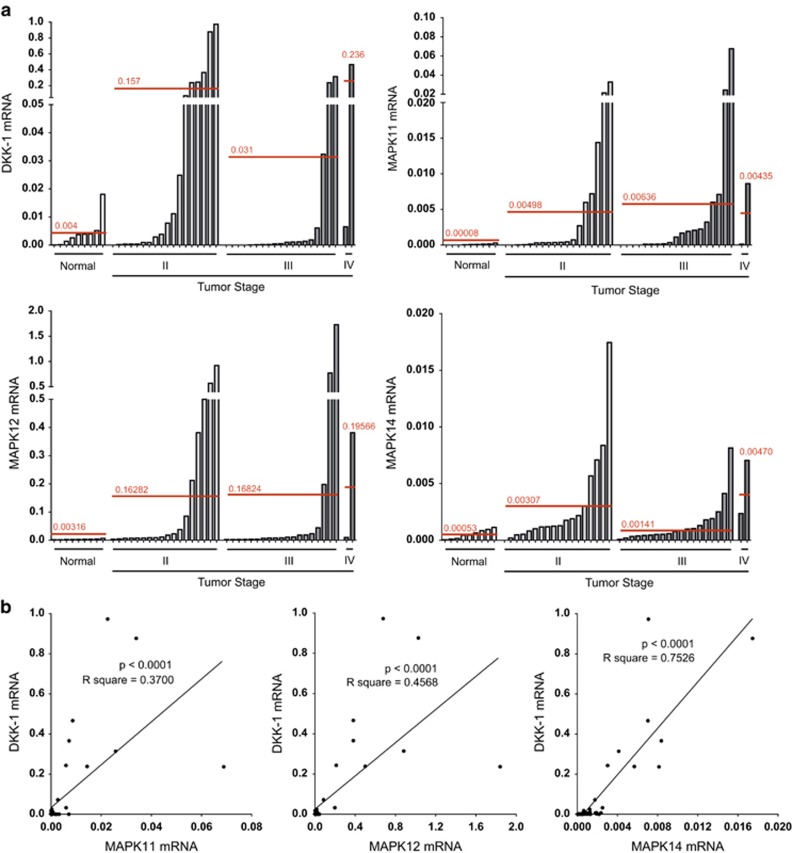
DKK-1 and p38 MAPK expression are correlated in prostate cancer. (**a**) A cDNA prostate cancer array was used to quantify DKK-1 and MAPK expression in prostate cancer. Expression levels were normalized to beta-actin. The intersecting red lines and values represent the mean expression in normal and progressive tumor stages. (**b**) Positive correlation of DKK-1 with each p38 MAPK isoform tested in prostate cancer cDNA samples. Correlation was calculated using Pearson's r correlation analysis and linear regression calculation

In order to ascertain whether regulation of DKK-1 by p38 MAPK has clinical relevance in human prostate cancer, a cDNA array of human prostate cancer samples was analyzed. A strong expression of both DKK-1 and p38 MAPKs was observed in all patients with progressive disease stages from II to IV, compared with an inherent low expression in healthy controls (Figure 6a). In addition, all investigated p38 MAPKs were positively correlated with that of DKK-1 in these samples (*P*<0.0001). In particular, MAPK14 expression shared the highest correlation with that of DKK-1 (Figure 6b).

## Discussion

Hormone-independent or androgen-resistant prostate cancer is prone to metastasize to the bone and requires more effective treatment options such as new secondary agents to combine with current treatment protocols.^32, 33^ Upon reaching the bone, the patient's prognosis remains poor, however, when the number of metastases are lower (<6) the prognosis is more favorable.^34^ Therefore, the identification of therapeutic targets and treatment options aimed at preventing and reducing metastatic progression are of principal importance. DKK-1 is proposed as such a target. It is acknowledged that DKK-1 can stimulate the growth of prostate cancer and metastasis, whereas inhibiting the osteoblastic drive of bone formation.^21, 35^ Currently, the efficacy of targeting DKK-1 in multiple myeloma is proving positive in the clinical setting,^36^ and although therapeutic targeting of DKK-1 may have translational potential in inhibiting the growth and metastasis of solid tumors,^37^ further research is required to understand the molecular mechanisms that are actively involved in DKK-1 regulation.

In this study, we have employed the use of various prostate cancer cell lines, which display varying degrees of osteolytic activity *in vivo*. The MDA-PCa-2b cell line has the lowest osteolytic potential, forming osteoblastic lesions *in vivo* by stimulating the differentiation and proliferation of osteoblasts through a Cbfa-1-dependent pathway.^38^ C4-2B cells promote mixed osteolytic and osteoblastic lesions *in vivo* by the expression of Wnts and BMPs, which directly promote osteoblastogenesis and indirectly promote osteoclastogenesis.^35, 39^ Similarly, DU145 cells also promote the formation of mixed lesions *in vivo*. This action has been shown to be mediated by enhancing the activation of pre-osteoblasts through ERK1/2 and STAT3 signal transduction pathways, simultaneously resulting in an increased RANKL expression, which activate osteoclast precursors and promote osteoclastogenesis.^40^ By contrast, PC3 cells display the most striking osteolytic phenotype *in vivo*, primarily inhibiting osteoblastogenesis by secreting DKK-1 and secreting matrix metalloproteinases, which directly degrade mineralized and non-mineralized bone.^35, 41^

We first verified that DKK-1 is overexpressed in the PC3 prostate cancer cells. This is in contrast to those prostate cancer cell lines that mainly form osteoblastic and mixed lesions *in vivo,*^35, 38, 39, 40^ and express low levels of DKK-1. When investigating the consequences of elevated DKK-1 secretion on the process of osteoblastogenesis, it is observed that higher levels of PC3-derived DKK-1 have greater inhibitory effects, suppressing the mRNA expression of osteoblastic markers like ALP, osteoactivin and OPG *in vitro*. This direct effect on osteoblast differentiation confirms one possible role of prostate-derived DKK-1 in the derivation of osteolytic lesions. Leading on from previous findings in multiple myeloma,^23^ this study aimed to investigate p38 MAPK as a regulator of DKK-1 in prostate cancer.

The bacterial antibiotic, anisomycin, is a potent activator of p38 MAPK^42, 43^ as shown by increased phosphorylation of p38 MAPK and HSP27 a specific downstream target.^44, 45^ In the prostate cancer cell lines investigated, this resulted in a potent increase of DKK-1 mRNA expression, most apparent in the osteolytic PC3 cells. Of note, treatment with p38 inhibitors suppressed this increase in DKK-1 mRNA expression and p38 MAPK/HSP27 protein phosphorylation, supporting the distinct role of p38 MAPK signaling as anisomycin itself also induces the JNK/SAPK signaling pathway.^46^ As shown by Thudi *et al.*,^47^ the upregulation of DKK-1 in the osteoblastic prostate cancer cell line Ace-1 altered the osteoblastic phenotype toward an osteolytic phenotype *in vivo.* This highlights a key role of the levels of the Wnt inhibitor DKK-1 in regulating the osteoblastic/osteolytic appearance of prostate cancer bone metastases. We show here that the activation of p38 MAPK signaling using anisomycin also mediates an increased DKK-1 expression in prostate cancer cell lines, which normally have low levels of DKK-1. Although the increases in DKK-1 mRNA expression are not to the same level of those observed in the untreated PC3 cells, they are indicative of a role of p38 signaling in defining the osteotropic signature of prostate cancer cells.

When used to target p38 MAPK in solid malignancies, the small molecule inhibitors, LY2228820 and SB202190, had promising antitumor effects in preclinical studies,^48, 49^ and their therapeutic potential is being currently investigated in clinical trials (NCT01393990, NCT01663857). Small molecule inhibitors of p38 MAPK display varying potencies of inhibition with regard to the individual MAPK isoforms (according to the supplier). Although our results show that three such inhibitors had suppressive effects on DKK-1 expression, some more potent than others, it is difficult to differentiate further the role of the individual isoforms.

To elucidate further the association between DKK-1 and individual p38 MAPK isoforms, PC3 cells were transfected with siRNA directed against MAPK11, MAPK12 and MAPK14. Of note, MAPK11 knockdown negatively regulated DKK-1 expression for all three siRNAs used, whereas MAPK12 had less of an effect with only two siRNAs showing a mild suppression of DKK-1 and only one of the siRNAs targeting MAPK14 having a significant negative effect on DKK-1 expression. Furthermore, when using the most potent siRNA per MAPK isoform, MAPK11 has the most suppressive effect on the functional secretion of the DKK-1 protein as detected by ELISA, followed by MAPK14. MAPK12 had no effect on secreted DKK-1 protein levels.

Here, we show that both MAPK11 and MAPK14 regulate DKK-1 to different degrees. However, it is important to consider other known functions of these isoforms before determining a preference for inhibiting one over the other. MAPK11 activity in breast cancer cells leads to an increased secretion of monocyte chemotactic protein-1, which results in enhanced osteoclastogenesis and bone resorption.^28^ Therefore, targeting MAPK11 activity could be desirable to not only promote osteoblastogenesis, but also to inhibit osteoclastogenesis and maintain bone integrity at sites of osteolytic bone metastases. On the other hand, MAPK14 has been shown to be active in osteoblasts and to be required for maintaining differentiation and physiological function.^50^ This would endorse MAPK11 further, as being a more suitable target for an *in vivo* approach in future.

By suppressing DKK-1 expression directly using siRNA, or indirectly by targeting p38 MAPK, we achieved rescue effects on the normal suppression of ALP and osteoactivin by PC3 supernatant in osteoblast experiments. The data also suggest that the use of p38 MAPK inhibitors could have a positive effect on bone homeostasis by exerting bone protective effects in patients with prostate cancer where intensified levels of DKK-1 within prostate cancer metastases have been previously shown to correlate with poorer outcome and survival.^21^ As discussed earlier, caution must be used with regard to inhibition of MAPK14 and direct effects on osteoblasts and further research is warranted to exclude undesired effects on bone formation. In this context, targeting MAPK11 would appear to be a more attractive option to selectively target DKK-1. However, to our knowledge inhibitors selectively targeting this isoform are not available at the moment.

An important observation that warrants discussion is that inhibiting the expression or activity of p38 does not reduce DKK-1 expression and secretion down to the levels first observed in the non-osteolytic prostate cancer cell lines. Hence, we cannot attribute the observed differences in DKK-1 expression between the different lines exclusively to differential MAPK signaling. Although we have shown than p38 MAPK is a significant regulator of DKK-1 in prostate cancer, there are other known regulators of DKK-1 expression in other cancer types such as JNK signaling induced by oxidative stress in multiple myeloma,^51^ and Wnt signaling, which negatively regulate DKK-1 in a feedback loop involving the beta-catenin/TCF pathway in prostate and liver hepatocellular carcinomas.^52^

In line with previous results,^20^ we confirmed increased DKK-1 expression levels in prostate cancer tissue by analyzing a cDNA array. P38 MAPKs were also increased in prostate cancer tissues compared with normal controls and furthermore, a correlation between p38 MAPKs and DKK-1 was evident. In the case of these clinical samples, MAPK14 showed the strongest correlation with DKK-1 expression. The overall correlation between the canonical Wnt inhibitor DKK-1 and p38 MAPKs may not in fact be that surprising. Like Wnt,^9^ p38 MAPK signaling is essential in the development of the skeleton and continued bone homeostasis in the adult.^53, 54^ The cross-talk between p38 MAPK and canonical Wnt signaling has also been clearly shown in a mouse model of teratocarcinoma.^55^ However, despite the strength of our own observations, they are potentially limited because of a small sample number of only 48 patients. Increasing the sample number in the future would further substantiate this data.

In summary, the p38 MAPK isoform, MAPK11 correlates with DKK-1 expression in different stages of prostate cancer and is the main p38 MAPK isoform regulating DKK-1 expression in osteolytic prostate cancer cells *in vitro*. Future research focusing on the MAPK11 isoform independently may develop this information and advance therapeutic regimes for treating osteolytic prostate metastases.

## Materials and Methods

### Cell culture

Prostate cancer cells (PC3, MDA-PCa-2b, DU145 and C4-2B) were purchased from ATCC (Manassas, VA, USA). In osteoblast experiments, the murine myoblast cell line C2C12 was used in association with control L-cells and WNT3A-L-cells; these cell lines were a kind gift from Dr. Michael Stock (University of Erlangen, Germany). Prostate cancer cells were cultured in RPMI 1640 medium (Gibco, Life Technologies GmbH, Darmstadt, Germany), apart from the MDA-PCa-2b cells, which were cultured in BRFF-HPC1 medium (Athena Enzyme Systems, Baltimore, MD, USA). C2C12 and L-cells were cultured in DMEM/F-12 (Gibco, Life Technologies GmbH). Cell cultures were maintained in a humidified atmosphere at 37 °C in 5% CO_2_–95% air and all culture medium conditions were supplemented with 10% (20% for MDA-PCa-2b) fetal calf serum supreme (FCS) (FBS; Biochrome, Berlin, Germany) and 1% penicillin/streptomycin (P/S) (Gibco, Life Technologies GmbH). Cells gifted from another institution and not purchased from ATCC were transferred and accepted under the ethical guidelines of both the providing institution and those of our own institution. The genetic authenticity of each cell line was verified at the DSMZ (German Collection of Microorganisms and Cell Cultures) where short tandem repeat profiling was matched with known profiles.

### Reagents and antibodies

P38 inhibitors were purchased as follows: LY228820 and SB202190 from Selleck Chemicals (Houston, TX, USA); Doramapimod from Medichem Express (Princeton, NJ, USA) and dissolved in DMSO. Anisomycin was purchased from Enzo Life Sciences (Farmingdale, NY, USA) and also solved in DMSO. Primary antibodies were purchased from the following providers: anti-DKK-1 (AF1096), anti-p38α (AF8691) and anti-p38γ (AF1347) from R&D Systems, Inc. (Minneapolis, MN, USA); anti-HSP27 (#2402), anti-p-HSP27 (#2405), anti-p38 (#2121) and anti-p-38 (#9211) from Cell Signaling Technology, Inc. (Beverly, MA, USA); anti-GAPDH (#5G4) from HyTest Ltd (Intelligate, Turku, Finland). Secondary HRP-conjugated antibodies: anti-goat IgG (HAF109), anti-mouse IgG (HAF007) and anti-rabbit IgG (HAF008) where purchased from R&D Systems, Inc. Control antibody for *in vitro* experiments: polyclonal goat IgG (AB-108-C) from R&D Systems, Inc. Recombinant bone morphogenetic protein 2 (BMP-2) was purchased from Peprotech (Rocky Hill, NJ, USA) and solved in water containing 0.1% bovine serum albumin (BSA) as the carrier protein.

### RNA isolation, reverse transcription and real-time polymerase chain reaction

The High-Pure RNA extraction kit (Roche Applied Science, Mannheim, Germany) was used to isolate RNA following the manufacturer's protocol. Briefly, 500 ng of purified RNA was reverse transcribed using Superscript II (Life Technologies, Darmstadt, Germany) and underwent SYBR green-based real-time polymerase chain reaction using a standard protocol (Applied Biosystems, Carlsbad, CA, USA). Primer sequences were as follows: 5'-CCAACCGCGAGAAGATGA-3' hu ACTB sense, 5'-CCAGAGGCGTACAGGGATAG-3'; hu ACTB antisense, 5'-GCCCAATACGACCAAATCC-3'; hu DKK-1 sense, 5'-AGCACCTTGGATGGGTATTC-3'; hu DKK-1 antisense, 5'-CACACTTGACCTTCTTTCAGGAC-3'; mu ACTB sense, 5'-GATCTGGCACCACACCTTCT-3' mu ACTB antisense, 5'-GGGGTGTTGA;AGGTCTCAAA-3' mu ALP sense, 5'-CTGGTGGCATCTCGTTATCC-3'; mu ALP antisense, 5'-CTACTTGTGTGGCGTGAAGG-3' mu OA sense 5'-AATGGGTCTGGCACCTACTG-3'; mu OA antisense, 5'-GGCTTGTACGCCTTGTGTTT-3'; mu OPG sense, 5'-CCTTGCCCTGACCACTCTTA-3' mu OPG antisense, 5'-GCACGTTCAATTCCTGGTTTAC-3'; hu MAPK11 sense, 5'-TCACAGTCCTCGTTCACAGC-3'; hu MAPK11 antisense, 5'-CAGGCAAGACGCTGTTCAAG-3'; hu MAPK12 sense, 5'-TGGTCAGGATAGAGGCAAAATC-3'; hu MAPK12 antisense, 5'-CGACTTGCTGGAGAAGATGC-3' hu MAPK 14 and 5'-GGCACAAAGCTGATGACTTC-3'; hu MAPK14 antisense. PCR cycling program ran at 50 °C for 2 min and 95 °C for 10 min followed by 40 cycles with 95 °C for 15 s and 60 °C for 1 min. The melting curve was assessed at 95 °C for 15 s, 60 °C for 1 min and 95 °C for 30 s. The ΔΔCT method was used to calculate the results, which are presented as the x-fold increase relative to the housekeeping gene (*β*-actin or m*β*-actin) or as a percentage of control.

### DKK-1 ELISA

The human DKK-1 ELISA was purchased from Biomedica (Vienna, Austria) and performed according to the manufacturer's instructions. Prostate cancer cell supernatants were prediluted at a ratio of 1 : 200 as determined by pretesting.

### Assessment of Wnt3a-induced osteoblastic differentiation in C2C12 cells

In the presence of Wnt3a, the murine myoblast cell line, C2C12, differentiate along the osteoblast lineage and are therefore a commonly used model to assess osteoblastogenesis. C2C12 cells were cultured for 72 h in the following supernatant combination: 10% medium from transfected L-cells overexpressing Wnt3a (control L-cell supernatant serving as the control), 75% unconditioned medium (5% FCS) and 15% untreated PC3 or pre-conditioned PC3 supernatants. Recombinant BMP-2 was also added at a concentration of 200 ng/ml to potentiate the induction of ALP and allow detection of enzymatic activity. Pre-conditioned PC3 supernatant was collected after siRNA knockdown or treatment with the p38 MAPK inhibitor LY2228820. Pre-treating PC3 cells with LY2228820 was performed to minimize direct effects of the inhibitors on C2C12 cells. This was achieved by culturing the PC3 cells with the inhibitor at a concentration of 10 *μ*M for 6 h, performing a fresh medium change and then collecting the supernatant after 18 h. Supernatant from PC3 cells transfected with siRNA was collected 72 h post transfection, including a fresh medium change at 24 h. C2C12 RNA was then isolated and assessed for ALP and osteoactivin expression by qRT-PCR. For detection of ALP activity, cells were washed in phosphate-buffered saline and lysed in 90 *μ*l of 10 mM Tris-HCl pH 8.0, 1 mM MgCl_2_ and 0.5% Triton X-100. After scraping, cell lysates were then transferred to 1.5 ml Eppendorf tubes, vortexed for 30 s and allowed to rest on ice for 20 min. The cell lysate was then centrifuged at 20 000 × *g* for 20 min at 4 °C. Supernatant was removed and 10 *μ*l aliquots were incubated with 90 *μ*l ALP substrate buffer (100 mM diethanolamine, 150 mM NaCl, 2 mM MgCl_2_ and 2.5 g/ml p-nitrophenylphosphate) for 30 min at 37 °C. The resulting absorbance at 410 nm was measured via spectrometer and normalized to total protein concentration measured by the bicinchoninic acid method.

### siRNA transfection

Sub-confluent PC3 cells in six-well dishes were transfected with the following siRNAs using Dharmafect (Thermo Scientific, Waltham, MA, USA): DKK-1 siRNA ID#s: s22723 and s22721 (Ambion, Life Technologies, Carlsbad, CA, USA); MAPK11 siRNA ID#s: MAPK11HSS183382, MAPK11HSS183383 and MAPK11HSS183384 (Invitrogen, Life Technologies, Carlsbad, CA, USA); MAPK12 siRNA ID#s: MAPK12HSS109466, MAPK12HSS109467 and MAPK12HSS109405 (Invitrogen, Life Technologies, Carlsbad CA, USA); MAPK14 siRNA ID#s: s3585, s3586 and s3587 (Ambion, Life Technologies, Carlsbad, CA, USA). Per six-well transfection, 100 nM siRNA were diluted in 50 *μ*l of OPTI-MEM and 2 *μ*l (should be 100 nM amount as varied) of DharmaFECT (Invitrogen) in 100 *μ*l of OPTI-MEM. SiRNA and DharmaFECT dilutions were incubated at room temperature for 5 min. The diluted siRNA was then combined with the diluted DharmaFECT at a ratio of 1 : 2, and incubated at room temperature for 20 min. Cells were washed twice with HBSS and medium replaced with 850 *μ*l of OPTI-MEM supplemented with 10% FCS. In all, 150 *μ*l of the siRNA and DharmaFECT mixture was then introduced drop-wise to the cells. After 5 h, the DharmaFECT mixture was replaced with the normal culture medium containing both FCS and P/S. The cells were further cultured for 24 h before supernatant was collected and cells lysed for either protein or RNA analysis.

### Wnt signaling assay

C2C12 cells were seeded at a concentration of 15 × 10^3^ cells per well, in 48-well plates and transfected with the Cignal TCF/LEF Reporter Assay kit (CCS-018L) (Qiagen, Hilden, Germany) to assess the activation of the TCF/LCF Wnt promotor. Briefly, 123 ng/cm^2^ of the promotor construct was transfected using the FuGENE HD Transfection Reagent (Promega, Madison, WI, USA) according to the manufacturer's protocols. After 24 h, C2C12 cells were treated with Wnt3a-containing L-cell medium and prostate cancer cell supernatants as indicated. Luciferase activity was assayed 24 h post treatment using the Dual Luciferase Reporter Assay kit (Promega) as instructed by the manufacturer.

### Immunoblotting

The analysis of protein expression by western blot was performed by the protocol described previously.^29^ In short, following siRNA knockdown or p38 MAPK inhibitor treatment, PC3 cells were lysed and protein levels quantified. Protein samples of 20 *μ*g were loaded to 10–12% SDS-PAGE and separated by electrophoresis. The separated proteins were then transferred onto a 0.2 *μ*m nitrocellulose membrane. Blocking was performed in 5% nonfat-dry milk in Tris-buffered saline with 1% Tween-20 for 1 h. Membranes were washed in Tris-buffered saline with 1% Tween-20 and incubated overnight in 5% BSA in Tris-buffered saline with 1% Tween-20 containing the primary antibody. Membranes were washed before incubation for 1.5 h with the horseradish peroxidase-conjugated secondary antibody in 1% nonfat-dry milk in Tris-buffered saline with 1% Tween-20. After another washing step, the membranes were developed and protein visualized using Super Signal (Pierce, Bonn, Germany) enhanced chemiluminescence.

### Prostate cancer array

The Prostate Cancer cDNA array III was sourced from Origene (Rockville, MD, USA) and the supplier's protocol was followed to assess the expression of DKK-1 and p38 MAPK isoforms when normalized to beta-actin. The array contained 48 samples in total; 9 samples of normal prostate tissue and 39 samples of prostate cancer with a selection of pathological grades from II to IV and an average patient age of 60 years.

### Statistical analysis

Each experimental set-up was repeated a minimum of three times and using GraphPad Prism 6 (GraphPad Software, Inc., La Jolla, CA, USA), one-way analysis of variance was performed to evaluate the equality of the mean. Correlation was calculated using Pearson's r correlation analysis and linear regression calculation. Results are presented as a standard deviation of the mean and a *P*-value of <0.05 was considered statistically significant.

## Abbreviations

DKK-1: Dickkopf-1
MAPK: mitogen-activated protein kinase
ALP: alkaline phosphatase
OPG: osteoprotegerin
BMP-2: bone morphogenetic protein 2
ELISA: enzyme-linked immunosorbent assay
HSP27: heat shock protein 27

## Notes

The authors Lorenz C Hofbauer and Tilman D Rachner have received honoraria, unrestricted educational grants and research funding from the following companies: Amgen, Novartis and Merck. The remaining authors declare no conflict of interest.
