# Supplementary figures and images for: p38 MAPK regulates the Wnt inhibitor Dickkopf-1 in osteotropic prostate cancer cells
Source: Cell Death Dis. 2016 Feb 25;7(2):e2119–. doi: 10.1038/cddis.2016.32 (PMC4849158; doi:10.1038/cddis.2016.32)

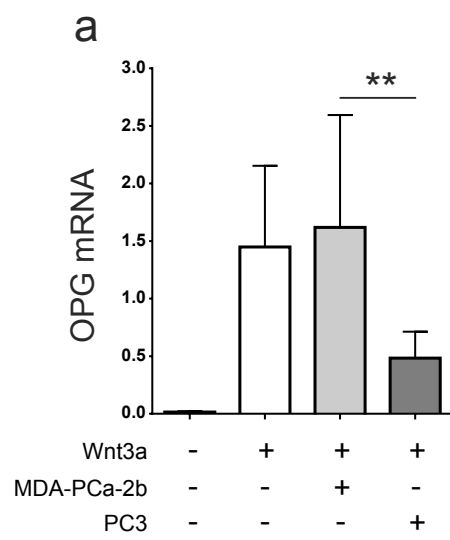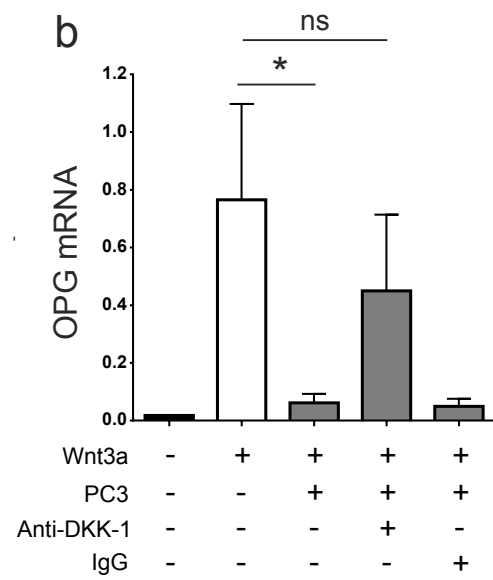

Supplementary Figure S1

Supplement: Supplementary Figure 1 [file cddis201632x1.pdf]

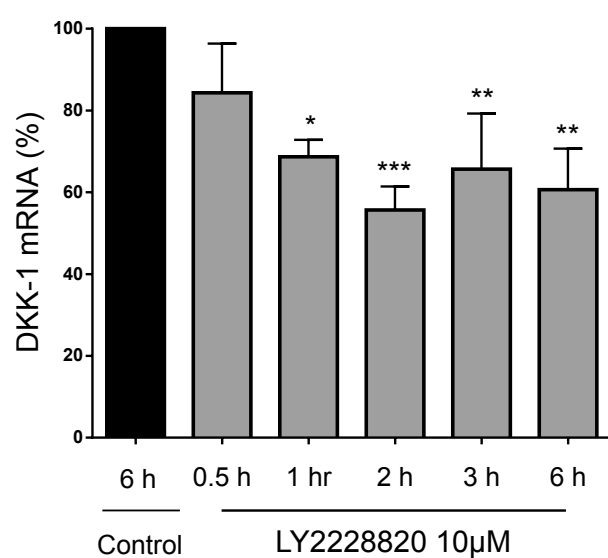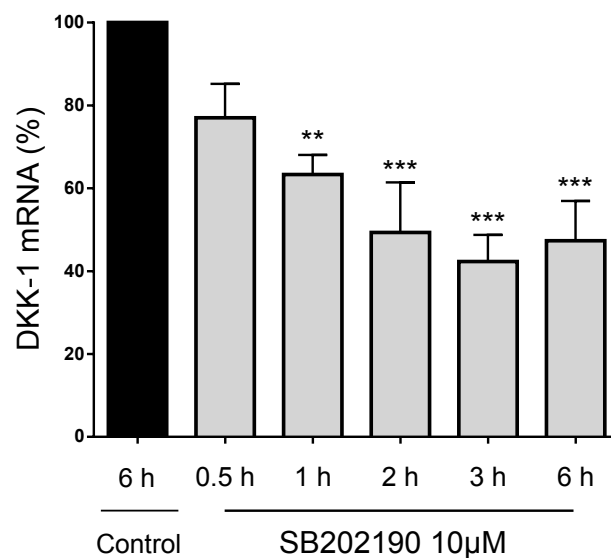

Supplementary Figure S2

Supplement: Supplementary Figure 2 [file cddis201632x2.pdf]

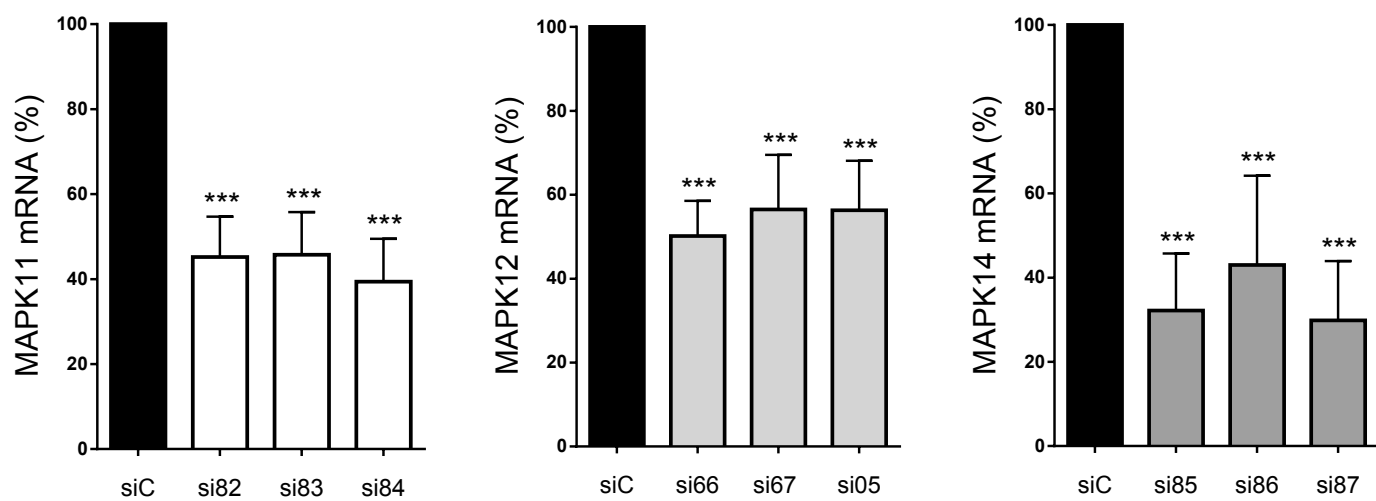

Supplementary Figure S3

Supplement: Supplementary Figure 3 [file cddis201632x3.pdf]

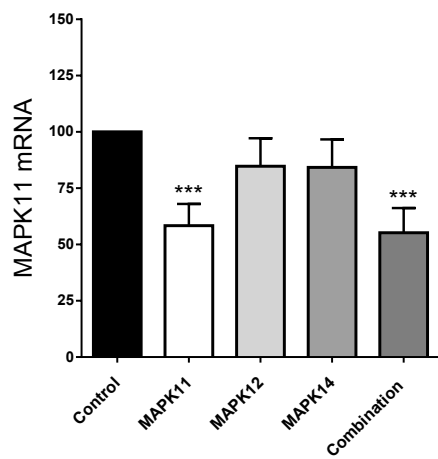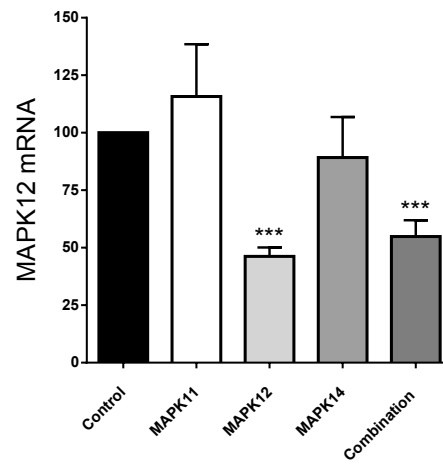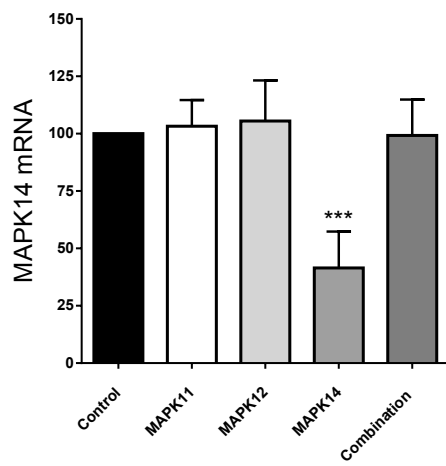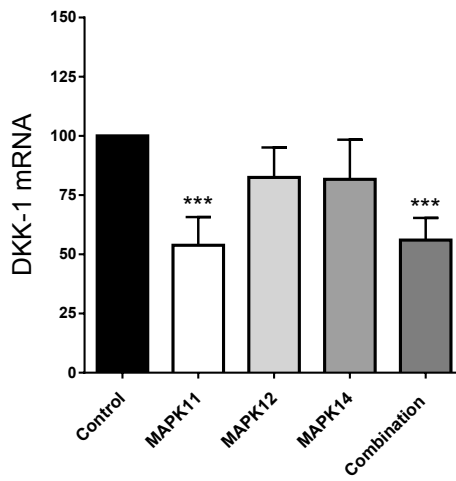

Supplementary Figure S4

Supplement: Supplementary Figure 4 [file cddis201632x4.pdf]
