# Supplementary material for: p38 MAPK regulates the Wnt inhibitor Dickkopf-1 in osteotropic prostate cancer cells
Source: Cell Death Dis. 2016 Feb 25;7(2):e2119–. doi: 10.1038/cddis.2016.32 (PMC4849158; doi:10.1038/cddis.2016.32)
Supplement: Supplementary Figure Legends [file cddis201632x5.doc]

**Supplementary figure legends**

**Supplementary Figure S1** Prostate cancer-derived DKK-1 inhibits Wnt3a induced OPG expression. The mRNA levels of the Wnt signaling marker OPG were assessed by qRT-PCR in C2C12 cells cultured in the presence of Wnt3a and prostate cancer supernatants (A) and the anti-DKK-1 antibody in the presence of Wnt3a and PC3 supernatant (B).

**Supplementary Figure S2** Inhibition of p38 MAPK signaling regulates DKK-1 in the prostate cancer cell line DU145. DU145 cells were treated for increasing time periods (hours) with small molecule inhibitors of p38 MAPK signaling; LY2228820 and SB202190.

**Supplementary Figure S3** mRNA expression of p38 MAPK isoforms following individual and combination knockdown in PC3 cells 24 hours post transfection, including the correlation with DKK-1 mRNA expression.

**Supplementary Figure S4** Knockdown of the individual p38 MAPK by siRNAs in PC3 cells. Each siRNA was validated by qRT-PCR.
